# Gastrodin Inhibits Virus Infection by Promoting the Production of Type I Interferon

**DOI:** 10.3389/fphar.2020.608707

**Published:** 2021-02-19

**Authors:** Yunlian Zhou, Mengyao Li, Tingyi Lv, Meixia Huang, Beilei Cheng, Yuanyuan Zhang, Jie Zhu

**Affiliations:** ^1^ Children’s Hospital, Zhejiang University School of Medicine, National Clinical Research Center for Child Health, Hangzhou, China; ^2^ Department of Clinical Laboratory, Zhejiang Hospital, Hangzhou, China

**Keywords:** gastrodin, macrophages, antivirus, IFN-I, IRF3

## Abstract

Type I interferon (IFN-I) plays a critical role in the antiviral immune response. However, viruses have developed different strategies to suppress the production of IFN-I for its own escape and amplification. Therefore, promoting the production of IFN-I is an effective strategy against virus infection. Gastrodin (GTD), a phenolic glucoside extracted from *Gastrodia elata* Blume, has been reported to play a protective role in some central nervous system -related diseases and is beneficial for the recovery of diseases by inhibiting inflammation. However, the effect of GTD on virus infection is largely unknown. Here we found GTD treatment increased the survival rate of mice infected with vesicular stomatitis virus (VSV) or herpes simplex virus-1 (HSV-1). The production of IFN-I was increased in GTD-treated mice or macrophages compared to the control group, during virus infection. Furthermore, the activation of interferon regulatory factor 3 (IRF3) was promoted by GTD in macrophages upon VSV and HSV-1 infection. Our results demonstrated that GTD could inhibit the VSV and HSV-1 infection by promoting the production of IFN-I in macrophages and might provide an effective strategy against virus infection.

## Introduction

Innate immunity is the first line of defense against virus infection (Corbett et al., 2020; Kucharski and Nilles, 2020; Wandernoth et al., 2020). Innate immune cells such as macrophages and dendritic cells are activated in response to virus infection, which depending on the detection of pathogen-associated molecular patterns (PAMPs) by germline-encoded pattern-recognition receptors (PRRs), including Toll-like receptors (TLRs), Retinoic acid-inducible gene I (RIG-I)-like receptors (RLRS), Nod-like receptors and DNA sensors such as cGAS, IFI16, and DDX41 (Oth et al., 2016; Eppensteiner et al., 2019). TLR3, 7, 8, and RLR members RIG-I and MDA5 detect the nucleic acid from RNA virus, such as severe acute respiratory syndrome coronavirus 2 (SARS-CoV-2), influenza A (H1N1), vesicular stomatitis virus (VSV) (El Chamy et al., 2008; Mu et al., 2019; Rehwinkel and Gack, 2020). TLR9 and DNA sensors recognize the viral DNA, after infection with DNA virus, such as herpes simplex virus-1 (HSV-1) and hepatitis B virus (HBV) (Unterholzner et al., 2010; Gao et al., 2013; Xie et al., 2018; Briard et al., 2020). Upon recognizing invading viruses, the PRRs recruit down-stream adaptors, including TRIF, MAVS and STING, which activate the down-stream kinases TBK1 and IKKε, leading to the activation of nuclear factor-kB and interferon (IFN) regulatory transcription factor (IRF)-3/7 and induction of the production of type I IFN (IFN-I) and proinflammatory cytokines (Chen et al., 2013; Chen et al., 2016; Gao et al., 2018; Liu et al., 2019).

IFN-I, mainly includes IFN-α and IFN-β, induces the expression of ISGs (IFN–stimulated genes), which can not only inhibit the virus at multiple stages from invasion to release, but also feedback regulate the expression of IFNs to play an indirectly antiviral effect (Shcheglovitova et al., 2016; Nganou-Makamdop et al., 2018; Li et al., 2019). It has been reported that IFN-I can suppress the SARS-CoV-2, VSV and HSV-1 infection (Hile et al., 2020; Lee and Shin, 2020; Walz et al., 2020). However, viruses such as H1N1 and HSV-1 can develop strategies to escape from the host immunity through inhibiting the production of IFN-I (Christensen et al., 2016; Wang et al., 2017; Lundberg et al., 2019). Therefore, improving the production of IFN-I is an effective strategy against virus infection.

Gastrodin (GTD), also named as 4-hydroxybenzyl alcohol-4-O-β-d-glucopyranoside, is a phenolic glycoside which is the glucoside of gastrodigenin (Liu et al., 2018). GTD is extracted from *Gastrodia elata* Blume and possesses the anti-apoptosis, and anti-inflammatory activities (Lv et al., 2020). As far, the research on GTD is mostly focused on its effect on the central nervous system (Zuo et al., 2016; Liu et al., 2018). Numerous studies have been reported that GTD possesses anti-oxidative stress, anti-inflammatory, and neuroprotective effects, which can relieve a broad range of CNS-related diseases, such as epilepsy, Alzheimer’s disease, Parkinson’s disease, affective disorders, cerebral ischemia/reperfusion. It has been reported that GTD acts as a protective molecular in LPS/GalN-induced fulminant hepatitis (Lv et al., 2020). GTD inhibits hepatocyte apoptosis through the inhibition of NLRP3 inflammasome and NF-κB activation, and the recovery of AMPK/ACC/autophagy (Sun et al., 2019). However, the effect of GTD in antiviral infection is largely unknown.

In our study, we found that GTD could inhibit the VSV and HSV-1 infection *in vivo* and *in vitro*. The production of IFN-I was also increased by GTD after VSV or HSV-1 infection. However, the expression of proinflammatory cytokines including tumor necrosis factor (TNF-α) and Interleukin 6 (IL-6) had no difference after treatment with GTD. We also found GTD promoted the activation of IRF3, thus facilitating the production of IFN-I. Our results demonstrated that GTD could inhibit the VSV and HSV-1 infection by promoting the production of IFN-I in macrophages and might provide an effective strategy against virus infection.

## Materials and Methods

### Mice, Cells and Reagents

C57BL/6J mice (6–8 weeks old) were purchased from SIPPR-BK Laboratory Animal Co., Ltd. (Shanghai, China). All mice were kept in the university laboratory animal center in an environment free of specific pathogens. All animal experiments were carried out following the protocol approved by the Animal Ethics Committee of Zhejiang University and conformed to institutional guidelines. Primary peritoneal macrophages were collected by peritoneal lavage from mice after intraperitoneal injection with thioglycolate (BD, Sparks, MD) for four days, and cultured in RPMI 1640 medium with 10% (vol/vol) fetal bovine serum (FBS). Antibodies specific for TBK1 (D1B4; 3504), TBK1 phosphorylated at Ser 172 (5483), IRF3 (4962), IRF3 phosphorylated at Ser396 (4947), IRF7 (4920), p-IRF7 (5184), p-JNK (9251), JNK (9252), p-p65 (3,033), p65 (8242), p-p38 (9215), p38 (9212), p-ERK (4370) and ERK (4695) were from Cell Signaling Technology. Anti-mouse IFNAR1 Ab (16-5945-025) was from eBioscience. GTD (HY-N0115) was purchased from MedChemExpress (MCE).

### Viruses and Viral Infection

VSV, HSV-1 and GFP-VSV are gifts provided by professor Wang Xiaojian (Zhejiang University, Hangzhou). Primary peritoneal macrophages (1 × 10^6^) were cultured in 12- well plate and infected with VSV at a multiplicity of infection (MOI) of 0.1, 1 or 10, VSV-GFP (MOI = 1) or HSV-1 (MOI = 10) at indicated time. For *in vivo* studies, mice were infected with VSV (1 × 10^8^ pfu/g) or HSV-1 (2 × 10^7^ pfu/g) by intraperitoneal injection.

### Tissue Culture Infective Dose (TCID_50_) Assay

This experiment was performed as we described previously (Chen et al., 2016). The snap-frozen lung, spleen, and brain liver tissues from mice were weighed (20 mg) and homogenized three times (5 s each) in 1 ml MEM, and then centrifuged at 1,600 g for 30 min, and the supernatants were collected. The supernatants from infected cells or tissues were serially diluted and infected on Vero cells for 1 h. The cells were covered with growth medium containing 0.6% low melting point agarose. After 16 h, plaques stained with 0.5% crystal violet (m/v) in 20% ethanol (v/v) were counted to calculate viral titer as previously described (Xing et al., 2011; Xing et al., 2013).

### Flow Cytometry

Primary peritoneal macrophages were infected with GFP-VSV for indicated time. Then cells were digested by trypsin and washed by PBS. After resuspended in PBS, cells were analyzed by flow cytometry (FACS).

### Enzyme Linked Immunosorbent Assay (ELISA)

The supernatants were collected from primary peritoneal macrophages or peripheral blood in the indicated experiments. The concentrations of IFN-α, IFN-β or TNF-α cytokine were detected with ELISA kits according to the manufacturer’s protocols (InvivoGen). The capture antibody was added to the plate, and incubated overnight at 4°C. The plate was washed three times using wash buffer, then added blocking buffer to each well and covered at room temp for 1 h. The samples or standards were added to the plate and incubated at room temp for 1 h after removing the blocking buffer. The plate was washed 3 times 5 min each, then added biotinylated detection antibody and incubated at room temp for 1 h. The enzyme conjugate was added to each well and incubated at room temp for 1 h after washing three times 5 min each. The plate was washed three times 5 min each, added substrate solution to each well and incubated at room temp for 30 min. The absorbance was measured by appropriate hardware and analyzed the data.

### Quantitative Real-Time PCR (qRT-PCR)

Total RNA was isolated from cells using TRIzol reagent (Takara) according to the manufacturer’s instructions. Single-stranded cDNA was generated from total RNA by using reverse transcriptase kit (Toyobo). SYBR Green master Rox (Roche) was used to quantitative Real-time reverse transcriptase-PCR analysis. qPCR primers were as follows:

VSV forward: 5′-ACG​GCG​TAC​TTC​CAG​ATG​G-3′;

VSV reverse: 5′-CTC​GGT​TCA​AGA​TCC​AGG​T-3′;

HSV-1 genomic DNA forward: 5′-TGG​GAC​ACA​TGC​CTT​CTT​GG-3′;

HSV-1 genomic DNA reverse: 5′-ACC​CTT​AGT​CAG​ACT​CTG​TTA​CTT​ACC​C-3′; mouse TNF-αforward 5′-AAG​CCT​GTA​GCC​CAC​GTC​GTA-3′;

mouse TNF- α reverse 5′-GGC​ACC​ACT​AGT​TGG​TTG​TCT​TTG-3′;

mouse IFN-βforward 5′-GGG​AGA​ACT​GAA​AGT​GGG​AAA-3′;

mouse IFN- β reverse 5′-ACC​TGC​AAG​ATG​AGG​CAA​AG-3′;

mouse IFN-αforward 5′- TGA​TGA​GCT​ACT​ACT​GGT​CAG​C-3′;

mouse IFN- α reverse 5′- GAT​CTC​TTA​GCA​CAA​GGA​TGG​C-3′;

mouse β-actin forward 5′-GAT​GAC​GAT​ATC​GCT​GCG​CTG-3′;

mouse β-actin reverse 5′-GTA​CGA​CCA​GAG​GCA​TAC​AGG-3′.

### Western Blot

To perform western blot analysis, the cells were treated with cell lysis buffer (CST, 9803) and inhibitor “cocktail” (Sigma, P8340). Protein concentration was measured by BCA assay (Pierce, 23235). An equal amount of protein was extracted from the sample for dodecyl sulfate-polyacrylamide gel electrophoresis. The protein was electrically transferred to the polyvinylidene difluoride membrane (Bio-Rad). Immunoblots were probed with the indicated antibodies. The protein bands were visualized by Pierce Chemiluminescence ECL Kit (Thermo).

### Lung Histology

The lungs from control or virus-infected mice were dissected and fixed in 10% phosphate buffered formalin, embedded in paraffin, then sectioned, stained by hematoxylin and eosin solution. The histological changes of sections were checked with an optical microscope.

### Statistical Analysis

A one or two -way ANOVA was used to determine the significance of difference between groups and the statistical significance of mouse survival study was generated and analyzed by Kaplan–Meier survival with GraphPad Prism software. *p* values of less than 0.05 were considered statistically significant.

## Results

### GTD Inhibits Virus Infection *in vivo*

**FIGURE 1:**
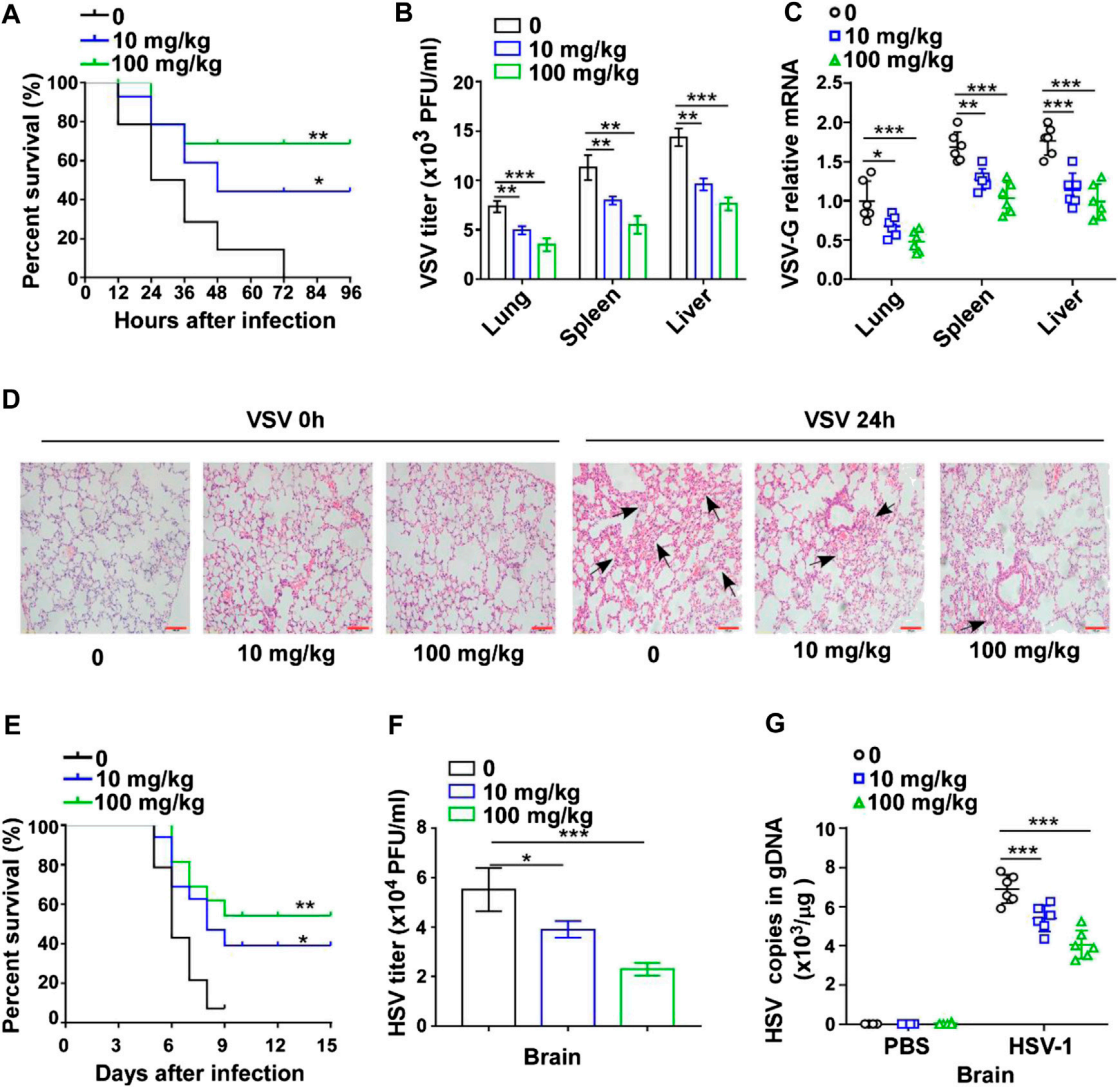
GTD protects mice from virus infection. **(A)** The survival curve of 8-week-old mice treated with DMSO, 10 mg/kg or 100 mg/kg GTD after intraperitoneal injection with VSV. **(B)** Determination of VSV loads in lung, spleen, and liver of mice after infection with VSV for 24 h. **(C)** qRT-PCR analysis of VSV transcripts in lung, spleen, and liver of mice in **(B)**. **(D)** Hematoxylin and eosin staining of lung sections from mice in **(B)**, and the arrow indicated the pathological damage part of lung. Scale bars, 100 μm. **(E)** The survival curve of 8-week-old mice treated with DMSO, 10 mg/kg or 100 mg/kg GTD after intraperitoneal injection with HSV-1. **(F)** Determination of HSV-1 loads in the brain of mice after infection with HSV-1 for 24 h. **(G)** qPCR analysis of HSV-1 genomic DNA copies in the brain of mice infected with HSV-1 for 24 h. The individual data points represent individual mice **(C,G)**. Data are presented as the mean ± SD. and are representative of three independent experiments. *p < 0.05; **p < 0.01; ***p < 0.001.

To explore the effect of GTD on virus infection in mice, the survival rate was initially recorded for 96 h after VSV challenge. Upon administration of VSV, the mice began to die at 12 h and the survival rate dropped to 0% at 72 h. In contrast, GTD treatment could prolong the survival time and enhance the survival rate of mice (40% in 10 mg/kg and 70% in 100 mg/kg) (Figure 1A). The VSV titers and VSV-G transcript levels in liver, spleen, and lung were significantly lower in GTD treated mice compared to control mice (Figures 1B,C). H&E analysis showed that less infiltration of inflammatory cells and pathological damage were observed in the lungs of GTD-treated mice after infection with VSV (Figure 1D). Next, we detected whether GTD plays a role in anti-DNA virus infection such as HSV-1. We found the survival rate of HSV-1 challenged mice was higher after treatment with GTD (Figure 1E). There were lower HSV-1 titers and HSV-1 gDNA copies in the brain of mice with GTD treatment (Figures 1F,G). These findings implied that GTD inhibits RNA virus and DNA virus infection *in vivo*.

### GTD Promotes IFN-I Production *in vivo*

**FIGURE 2:**
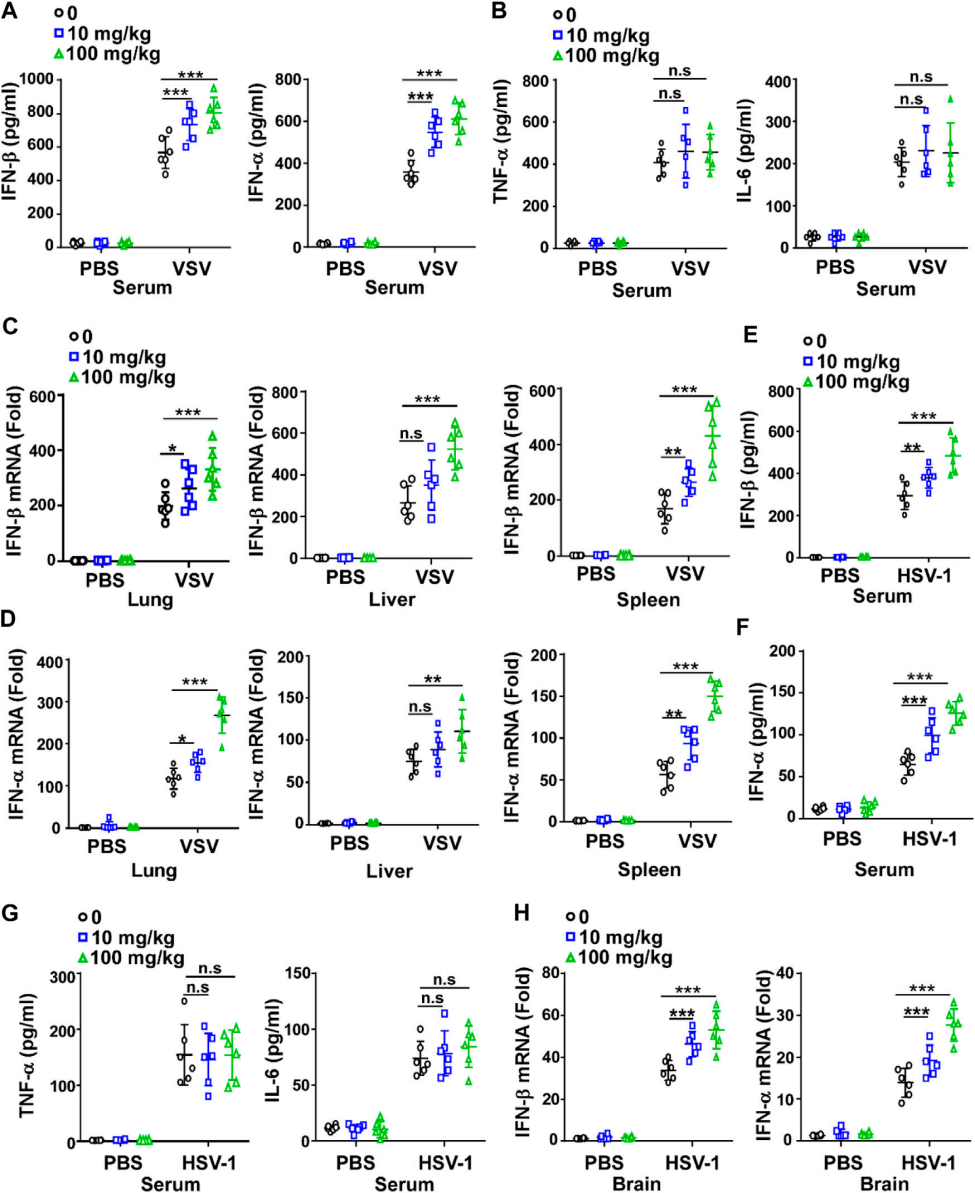
GTD enhances the production of IFN-I. **(A)** ELISA analysis of IFN-β and IFN-α in the serum of mice infected with VSV for 24 h **(B)** ELISA analysis of TNF-α and IL-6 in the serum of mice infected with VSV for 24 h. **(C,D)** qRT-PCR analysis of IFN-β **(C)** and IFN-α **(D)** transcription in lung, spleen, and liver of mice infected with VSV for 24 h. **(E,F)** ELISA analysis of IFN-β **(E)** and IFN-α **(F)** in the serum of mice infected with HSV-1 for 24 h. **(G)** ELISA analysis of TNF-α and IL-6 in the serum of mice infected with HSV-1 for 24 h. **(H)** qRT-PCR analysis of IFN-β and IFN-α transcription in the brain of mice infected with HSV-1 for 24 h. The individual data points represent individual mice **(A–H)**. Data are presented as the mean ± SD. and are representative of three independent experiments. *p < 0.05; **p < 0.01; ***p < 0.001.

Since IFN-I plays a critical role in the innate immune response against viruses, we investigated whether GTD could affect the production of IFN-I. The concentrations of IFN-β and IFN-α were higher in GTD treatment mice after VSV infection (Figure 2A). The concentrations of proinflammatory cytokines such as TNF-α and IL-6 were not significantly different between GTD treatment mice and control mice after VSV infection (Figure 2B). We also evaluated the mRNA levels of IFN-β and IFN-α in lung, liver and spleen tissues and found that the dose of 100 mg/kg for GTD treatment was able to increase expressions of IFN- β and IFN-α in liver, lung and spleen (Figures 2C,D). Upon HSV-1 infection, both 100 mg/kg and 10 mg/kg GTD were able to induce higher concentrations of IFN-β and IFN-α in the serum of mice (Figures 2E,F), but not the TNF-α and IL-6 inflammatory cytokines (Figure 2G). The mRNA levels of IFN-β and IFN-α were increased in the brain of mice after treatment with GTD and infection with HSV-1 (Figure 2H). These results indicated that GTD specifically enhances the production of IFN-I after RNA virus and DNA virus infection in mice.

### GTD Inhibits Virus Infection in Macrophages

**FIGURE 3:**
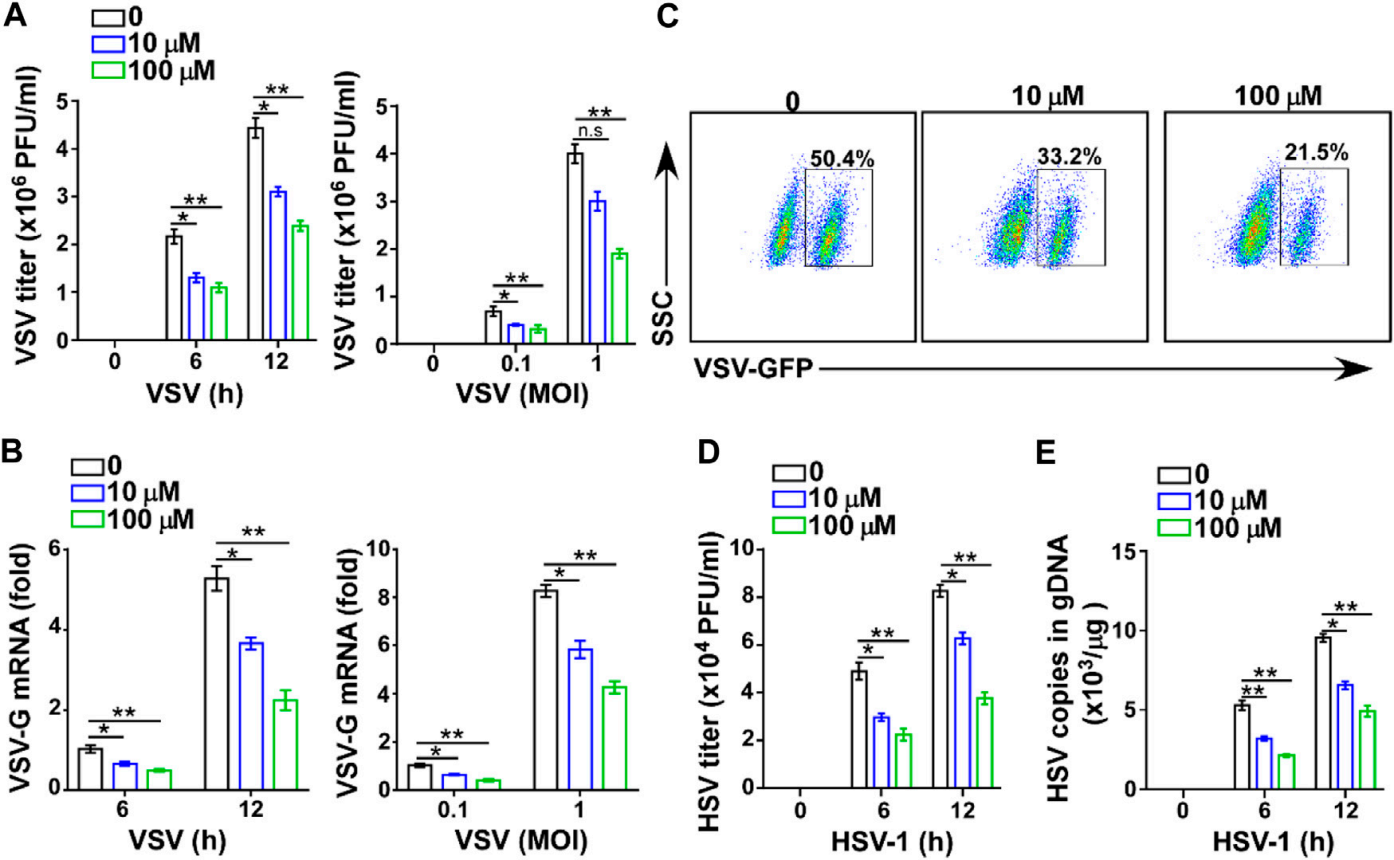
GTD inhibits virus infection in macrophages. **(A)** VSV loads analysis of macrophages pretreated with DMSO, 10 μM and 100 μM GTD followed by infection with VSV (MOI = 0.1 or 1) for indicated time. **(B)** qRT-PCR analysis of VSV transcripts in macrophage treated as in Figure 3A. **(C)** Flow cytometry analysis of GFP fluorescence intensity in macrophages pretreated with DMSO, 10 μM and 100 μM GTD, then infected with VSV for 12 h. **(D)** HSV-1 loads analysis of macrophages treated with DMSO, 10 μM and 100 μM followed by HSV-1 infection for 6 or 12 h. **(E)** qPCR analysis of HSV-1 gDNA in macrophages infected with HSV-1 for indicated time. Data are presented as the mean ± SD. and are representative of three independent experiments. *p < 0.05; **p < 0.01; ***p < 0.001.

To further confirm the effect of GTD in virus infection, we assessed the antiviral function of GTD in macrophages, which are at the front line and can produce large amounts of IFN-I to protect against fungi, bacteria, and virus infection (Chen et al., 2013; Channappanavar et al., 2016; Song et al., 2017). The low dose (10 μM) and high dose (100 μM) of GTD suppressed VSV titers when VSV infected macrophages *in vitro* (Figure 3A). To ensure the suppression of VSV infection, different amounts of VSV particles (0.1 and 1 MOI) were used to infect macrophages. Consistently, we detected that GTD enhanced viral clearance ability of macrophages under different counts of virus infection (Figure 3A). The VSV-G mRNA levels were also reduced while treatment with GTD (Figure 3B). By performing flow cytometry, we found that GTD-treated macrophages showed decreased GFP^+^ cells after infection with GFP-tagged VSV *in vitro* (Figure 3C). Correspondingly, GTD also reduced HSV-1 titers and HSV-1 gDNA in macrophages when infected with HSV-1 (Figures 3D,E). Taken together, these data suggested that GTD inhibits RNA and DNA viral susceptibility in macrophages.

### GTD Promotes IFN-I Production in Macrophages

**FIGURE 4:**
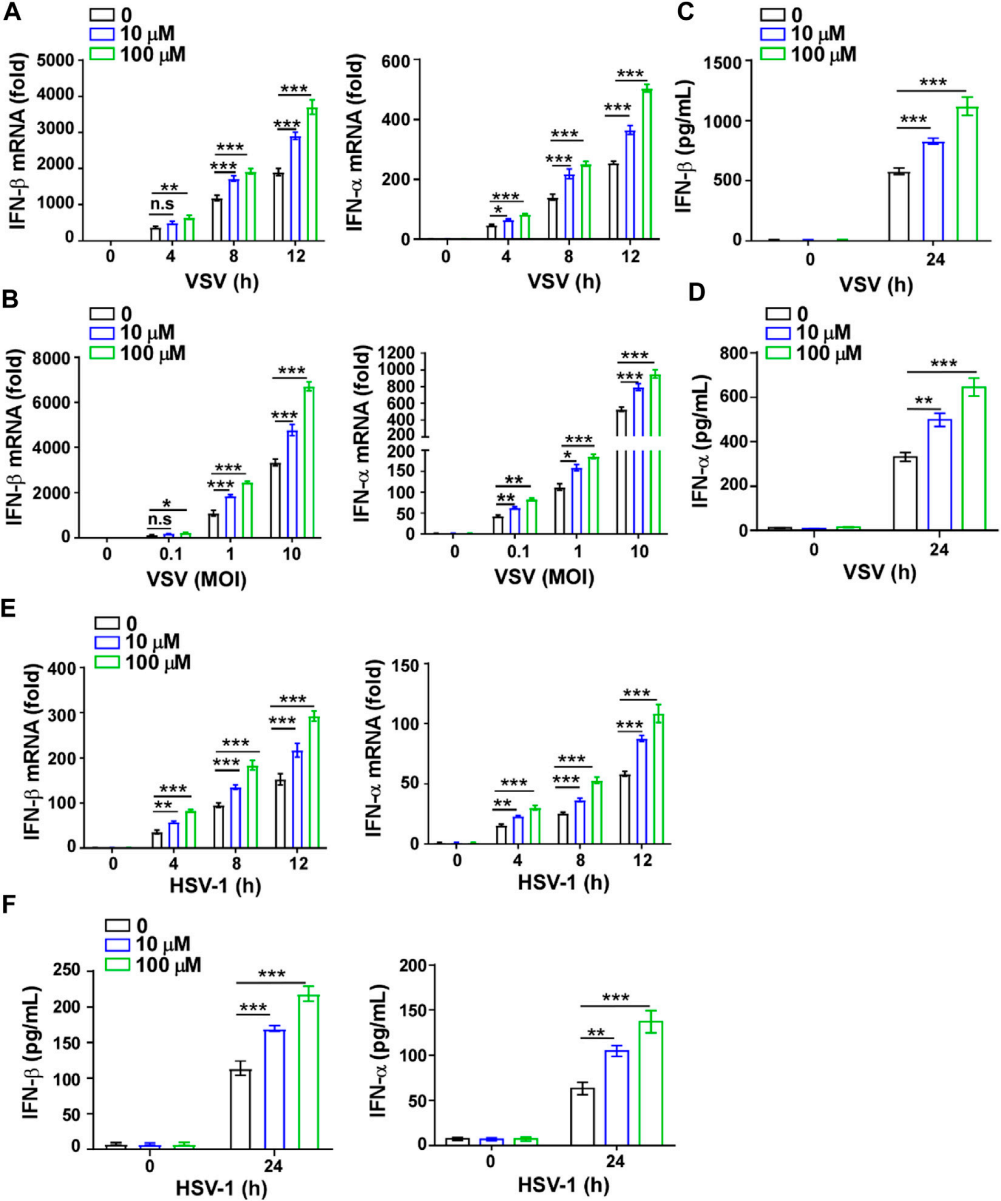
GTD promotes IFN-I production in macrophages. **(A)** qRT-PCR analysis of IFN-β and IFN-α in macrophages treated with 10 μM or 100 μM GTD followed by VSV infection for the indicated time points. **(B)** qRT-PCR analysis of IFN-β and IFN-α in macrophages treated with 10 μM or 100 μM GTD followed by infection with VSV (MOI = 0.1, 1 or 10) for 12 h. **(C,D)** ELISA analysis of IFN-β **(C)** and IFN-α **(D)** in macrophages treated with 10 μM or 100 μM GTD followed by infection with VSV for 24 h. **(E)** qRT-PCR analysis of IFN-β and IFN-α in macrophages treated with 10 μM or 100 μM GTD followed by infection with HSV-1 for the indicated time points. **(F)** ELISA analysis of IFN-β and IFN-α in macrophages treated with 10 μM or 100 μM GTD followed by infection with HSV-1 for 24 h. Data are presented as the mean ± SD. and are representative of three independent experiments. *p < 0.05; **p < 0.01; ***p < 0.001.

To further verify the effect of GTD on IFN-I, we also detected the levels of IFN-I and inflammatory factors in macrophages*.* The higher transcription levels of IFN-β and IFN-α but not TNF-α and IL-6 were detected in macrophages with GTD treatment after VSV infection (Figures 4A,B; Supplementary Figures S1A,B). Both doses of GTD could enhance the secretion of IFN-β and IFN-α, but not TNF-α and IL-6 in macrophages after virus infection (Figures 4C,D; Supplementary Figures S1C,D). Using HSV-1 infection experiments, we obtained the consistent results: GTD up-regulated IFN-β and IFN-α transcriptions when HSV-1 infected macrophages (Figure 4E). ELISA results demonstrated that IFN-β and IFN-α concentrations were higher in the supernatant of cultured-macrophages after HSV-1 infection (Figure 4F). There was no significant difference in the levels of TNF- α and IL-6 between GTD treatment and control group (Supplementary Figures S1E,F). These results indicated that GTD promotes the production of IFN-I in macrophages during VSV or HSV-1 infection.

### The Inhibition of Virus Infection by GTD was Dependent on IFN-I

**FIGURE 5:**
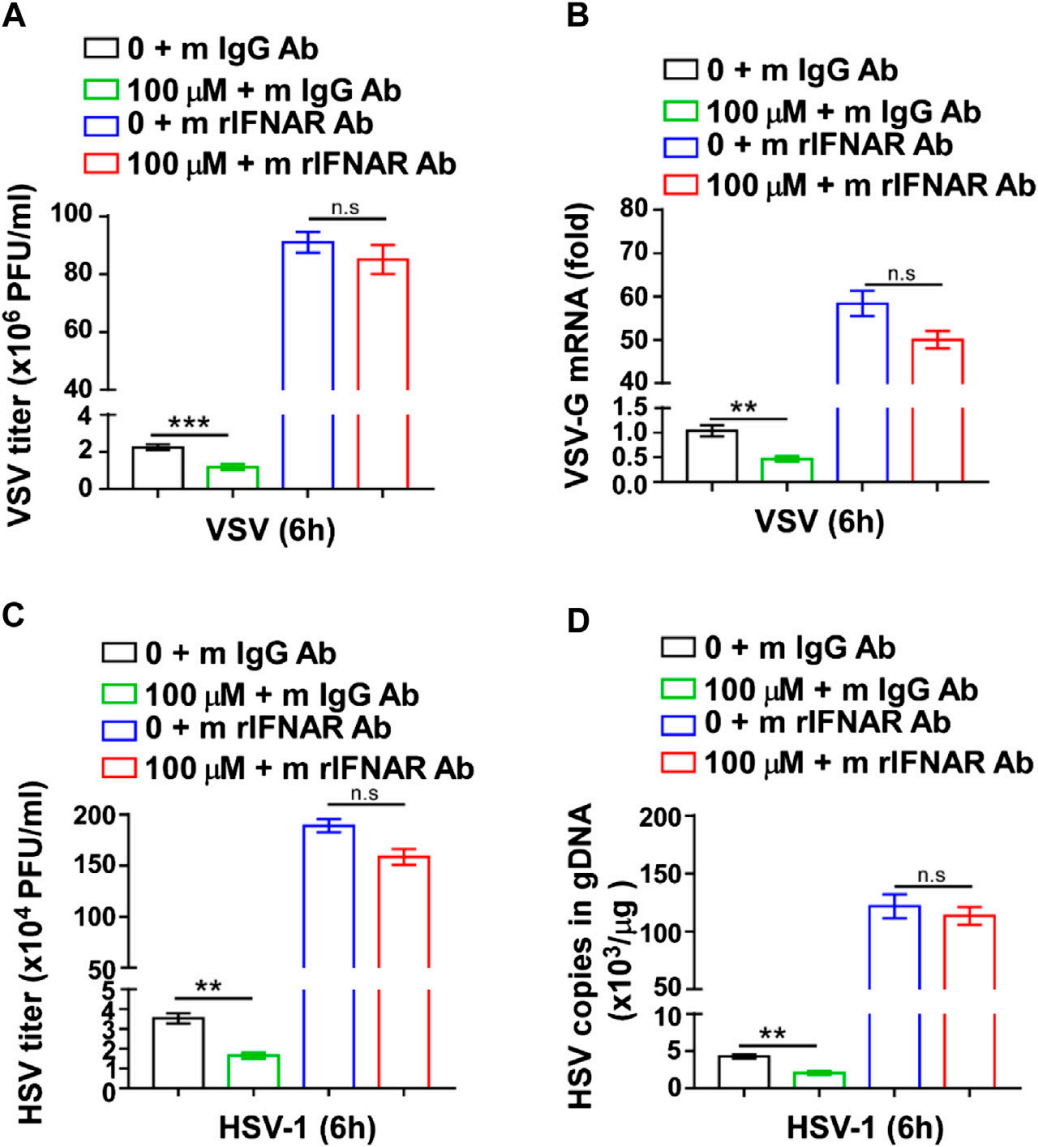
The inhibition of virus infection by GTD was dependent on IFN-I. **(A)** VSV loads analysis of macrophages pretreated with GTD or mIFNAR1 Ab followed by infection with VSV for 6 h. **(B)** qRT-PCR analysis of VSV transcripts in macrophages pretreated with GTD or mIFNAR1 Ab followed by infection with VSV for 6 h. **(C)** HSV-1 loads analysis of macrophages pretreated with GTD or mIFNAR1 Ab followed by infection with HSV-1 for 6 h. **(D)** qPCR analysis of HSV-1 copies in gDNA of macrophages pretreated with GTD or mIFNAR1 Ab followed by infection with HSV-1 for 6 h. Data are presented as the mean ± SD. and are representative of three independent experiments. **p < 0.01; ***p < 0.001.

To further verify whether the viral inhibitory effect of GTD was mediated by IFN-I, we used mouse IFNAR1(also known as IFN-α/β R1) Ab to interact with IFNAR1, block the IFN-I signal pathway and inhibit the expression of ISGs (Lindqvist et al., 2016). The VSV titer was significantly decreased in GTD treated macrophages after VSV infection (Figure 5A). However, the inhibition of VSV by GTD was not detected in macrophages after mouse IFNAR1 Ab treatment (Figure 5A). There was no significant difference in VSV-G mRNA levels between GTD and IFNAR1 Ab treated macrophages and control cells (Figure 5B). Furthermore, we did not observe the decreased HSV-1 titer and HSV-1 copies in gDNA by GTD in macrophages after treatment with IFNAR1 Ab (Figures 5C,D). Our results indicated that the inhibition of VSV or HSV-1 infection by GTD is dependent on IFN-I.

### GTD Enhances the Activation of IRF3

**FIGURE 6:**
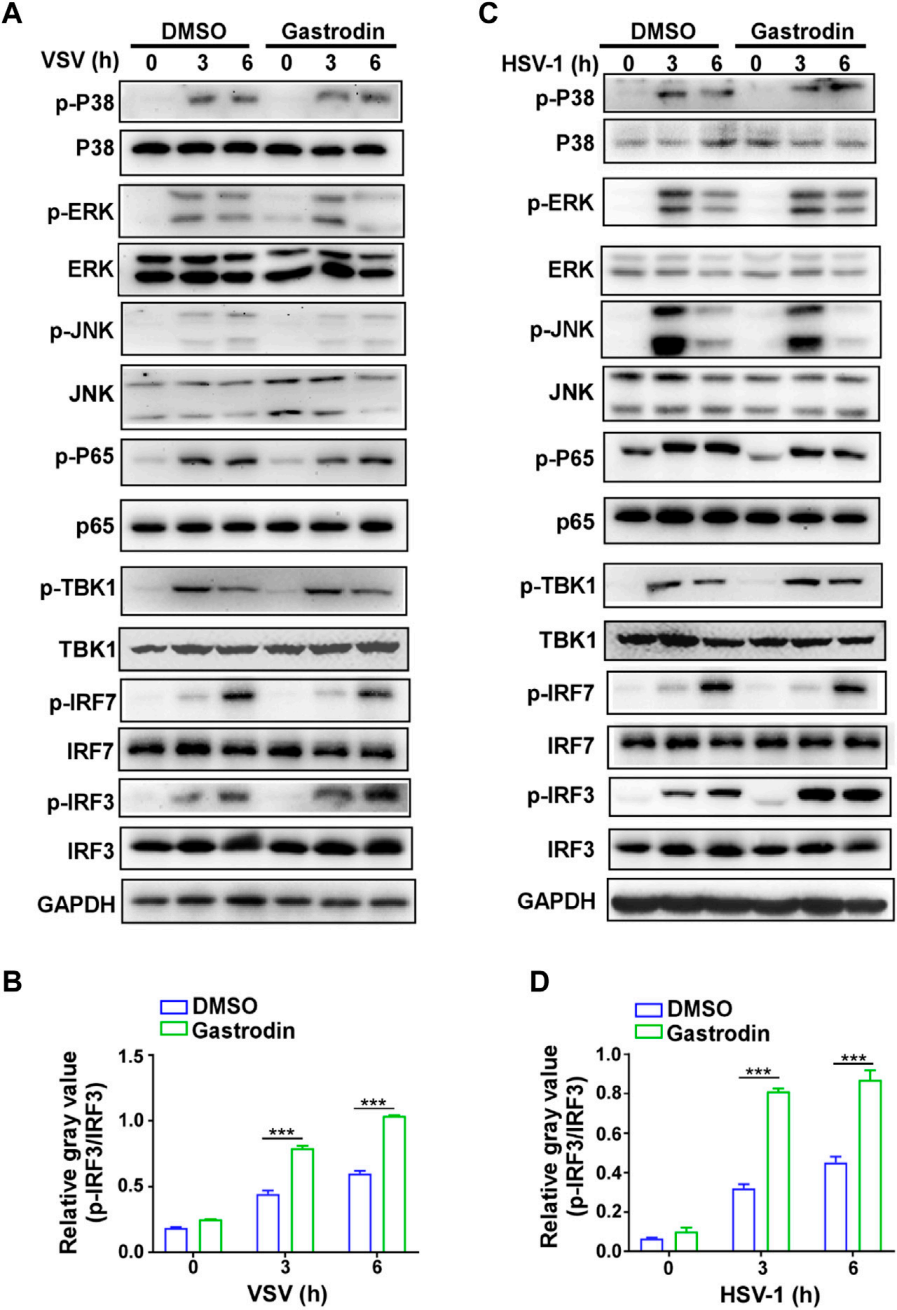
GTD promotes the activation of IRF3 in macrophages during virus infection. **(A)** Immunoblot analysis of phosphorylated (p-) or total proteins in lysates of macrophages treated with GTD after VSV infection. **(B)** Grayscale values of immunoblot analysis of phosphorylated (p-) or total proteins in lysates of macrophages treated as in Figure 6A. **(C)** Immunoblot figures of phosphorylated (p-) or total proteins in lysates of macrophages treated with GTD after HSV-1 infection. **(D)** Gayscale values of immunoblot analysis of phosphorylated (p-) or total proteins in lysates of macrophages treated as in Figure 6C. Data are presented as the mean ± SD. and are representative of three independent experiments. ***p < 0.001.

Next, we screened the downstream signaling pathways induced by virus in macrophages. The results showed that the activations of p38, ERK, JNK, p65, and TBK1 were not changed by GTD treatment (Figures 6A,B; Supplementary Figure S2A). Especially, phospho-IRF3 was highly induced by GTD in macrophages after VSV infection (Figure 6A). Consistently, the activation level of IRF3 is higher after HSV infection with GTD treatment (Figures 6C,D; Supplementary Figure S2B). Thus, these data implied that GTD promotes the production of IFN-β through up-regulating the IRF3 activation.

## Discussion

Previously, the research of GTD is mainly focused on the central neural system and its protective role on neuron is highly investigated. In some inflammation-related diseases such as lung injury, cardiac fibrosis and hepatic fibrosis, GTD also attenuates the severity of diseases through inhibiting the inflammatory factor release and oxidative stress (Shu et al., 2012; Zhao et al., 2015; Zhu et al., 2018; Liang et al., 2020). We speculate that GTD may also have an influence on other cell types, including innate immune cells, adaptive immune cells, and tissue structure cells, etc. Although, it has been reported that GTD derivatives have anti-influenza virus activity, the mechanism is untouched yet. Therefore, we used VSV and HSV-1 virus to detect the effect of GTD in the host-counteracting virus. Our data indicated that GTD could protect host from both DNA and RNA virus infection *in vivo* and *in vitro*. It has been reported that GTD treatment did not reduce the cell activity of macrophages, which indicated that GTD has no cell toxicity to macrophages (Jia et al., 2017). Taken together, GTD could be a safe and functional drug for antivirus.

IFN-I is induced by virus infection and plays a critical role in the host's innate antiviral response (Gruber, 2020; Israelow et al., 2020). However, viruses have evolved mechanisms to escape from the host immunity through inhibiting the production of IFN-I. For example, the VP1-2 protein of HSV-1 deubiquitinates STING to block type I interferon expression and promote brain infection (Bodda et al., 2020). The ICP0 protein of HSV-1 abrogates the production of IFN-I by mediating the degradation of STING (Kalamvoki and Roizman, 2014). VSV induces the expression of Siglec-G to promote the degradation of RIG-I and inhibit the production of IFN-I (Chen et al., 2013). Current research found that SARS-CoV-2 proteins such as ORF3a and ORF3b, inhibit the RIG/MDA5 signaling pathway or IFN-I signaling pathway to allow the virus to their escape (Sa Ribero et al., 2020). It has been reported that the severe and critically ill patients have a profoundly impaired IFN-I response with increased IL-6 and TNF-α compared with mild or moderate cases (Hadjadj et al., 2020). Therefore, improving the production of IFN-I is an effective strategy against virus infection. Our results showed that GTD promoted the expression of IFN-I, but not TNF-α and IL-6 during VSV or HSV-1 infection. Thus, we supposed that GTD might also inhibit the SARS-CoV-2 infection by enhancing the production of IFN-I. Further study needs to detect whether GTD can inhibit the SARS-CoV-2 infection by upregulating the production of IFN-I in clinical patients.

It has been reported that GTD inhibits the activation of NF-kB pathway in the LPS/GalN-induced fulminant hepatitis (Lv et al., 2020). Since GTD promotes the expression of IFN-I, which targets the common signaling pathway between DNA and RNA viruses, then we screened the common signaling pathway that produces IFN-I. The signaling pathway of NF-κB was not changed during the virus infection. These implied that the function of GTD depends on a specific environment. However, we found that the molecular mechanism involved in the upregulation of IFN-I by GTD is mediated by higher activated IRF3 which is a critical transcription factor for IFN-I production. GTD might target the activation of IRF3 through associating with molecular related with IRF3. Further research needs to investigate the underlying mechanisms involved in promoting the activation of IRF3 by GTD after virus infection in macrophages.

Our findings demonstrated that GTD may be an effective molecular that promotes IFN-I production through enhancing IRF3 activation to resist RNA and DNA virus infection. All these suggested that GTD is a potential candidate substance for fighting viruses.

## Data Availability

The original contributions presented in the study are included in the article/Supplementary Material, further inquiries can be directed to the corresponding authors.
