# Supplementary material for: Gastrodin Inhibits Virus Infection by Promoting the Production of Type I Interferon
Source: Front Pharmacol. 2021 Feb 19;11:608707. doi: 10.3389/fphar.2020.608707 (PMC7990098; doi:10.3389/fphar.2020.608707)
Supplement: Supplementary file 1 [file Image1.pdf]

Supplementary Data

Supplementary figure 1. GTD did not promote the production of TNF-α and IL-6 in macrophages.

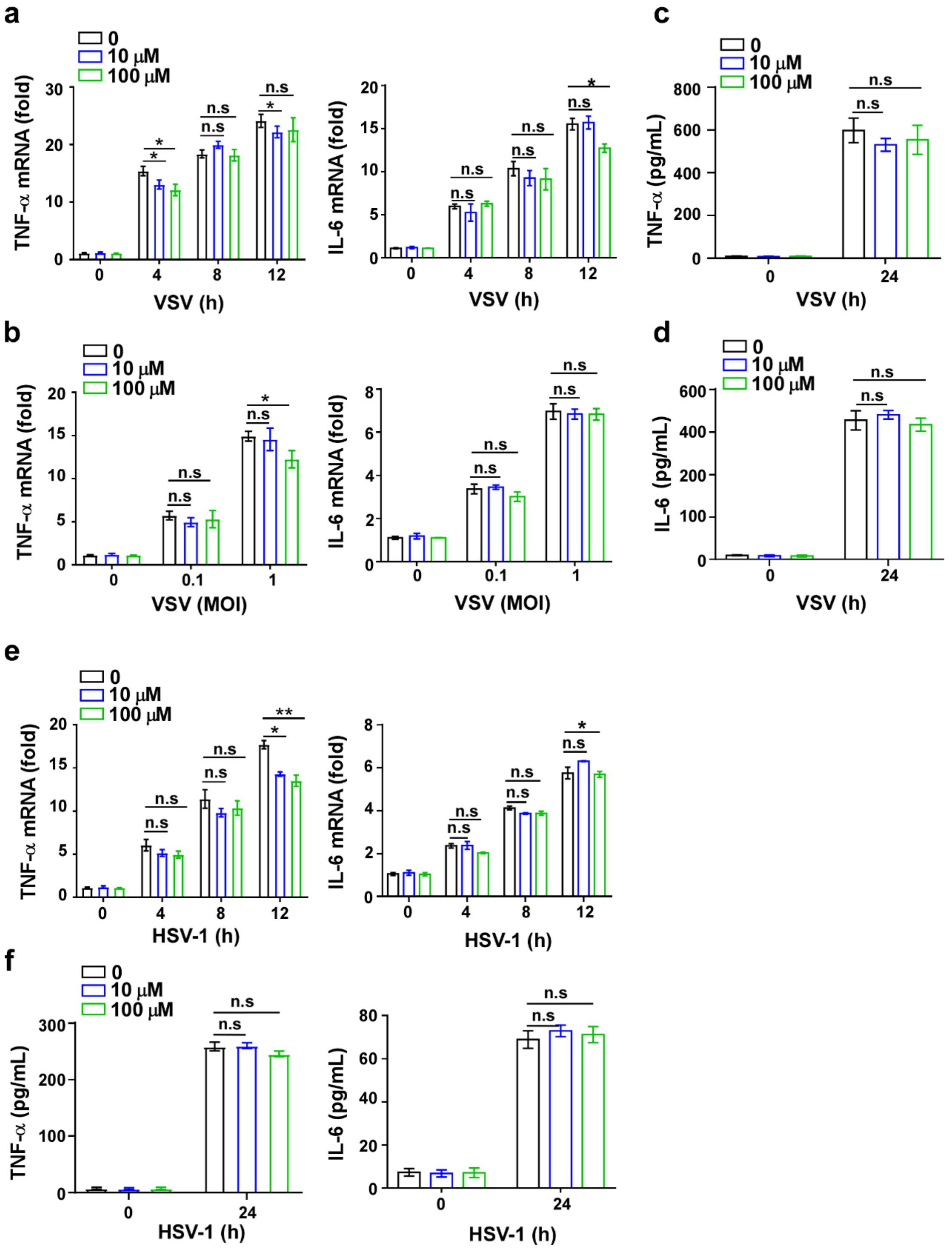

a. qRT-PCR analysis of TNF-α and IL-6 in macrophages treated with 10 μM or 100 μM GTD followed by infection with VSV for the indicated time. b. qRT-PCR analysis

of TNF-α and IL-6 in macrophages treated with 10 μM or 100 μM GTD followed by infection with VSV (MOI = 0.1 or 1) for 12 h. c-d. ELISA analysis of TNF-α (c) and IL-6 (d) in macrophages treated with 10 μM or 100 μM GTD followed by infection with VSV for 24h. e. qRT-PCR analysis of TNF-α and IL-6 in macrophages treated with 10 μM or 100 μM GTD followed by infection with HSV-1 for the indicated time. f. ELISA analysis of TNF-α and IL-6 in macrophages treated with 10 μM or 100 μM GTD followed by infection with HSV-1 for 24h. Data are presented as the mean ± SD. and are representative of three independent experiments. *$p < 0.05$.

Supplementary figure 2. GTD does not affect the activations of p38, ERK, JNK, p65, TBK1 and IRF7.

a

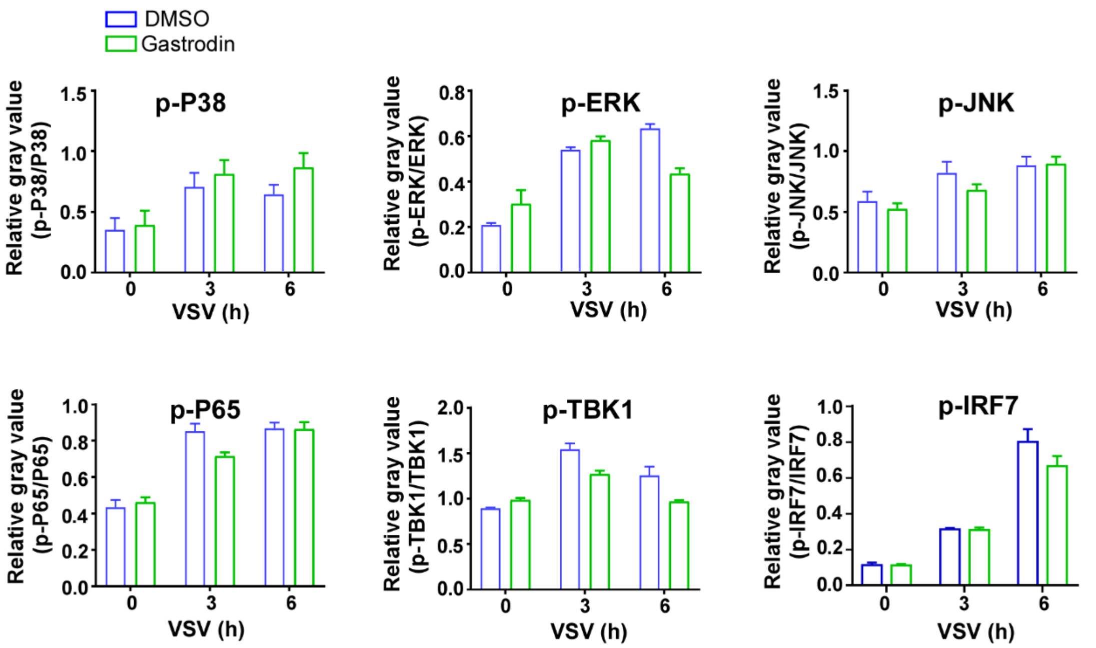

b

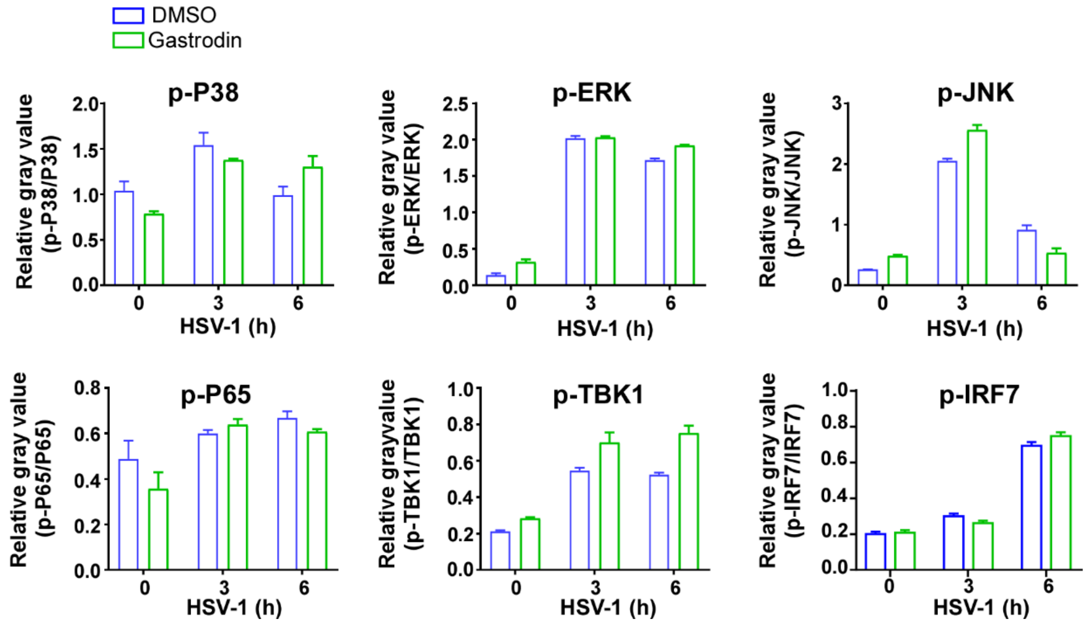

a. Grayscale values of immunoblot analysis of phosphorylated (p-) or total proteins in lysates of macrophages treated as in figure 6a. **b.** Grayscale values of immunoblot analysis of phosphorylated (p-) or total proteins in lysates of macrophages treated as in

figure 6c. Data are presented as the mean ± SD. and are representative of three independent experiments.
